# Neural correlations in the electrosensory lateral line lobe of the weakly electric fish, *Apteronotus leptorhynchus*: analysis of multi-channel recordings

**DOI:** 10.1186/1471-2202-15-S1-P190

**Published:** 2014-07-21

**Authors:** Teerawat Monnor, Michael G Metzen, Maurice J Chacron

**Affiliations:** 1 Department of Physiology, McGill University, Montreal, QC, H3G 1Y6, Canada; 2 Department of Physics, McGill University, Montreal, QC, H3G 1Y6, Canada

## 

It is recognized that perception and behavior result from the activities of large neural ensembles. As such, it is key to understand the mechanisms that give rise to correlated activity in the brain. However, correlated activity is highly plastic as it is regulated during specific behavioral contexts. In this work, we aim to understand how activation of neural circuits can shape correlated activity by using the weakly electric fish, *Apteronotus leptorhynchus*. We performed multi-channel recordings in the electrosensory lateral line lobe, which benefits from well-characterized neural architecture. First, a spike-sorting algorithm was applied on the recorded signals to extract neural units. Then, correlated activity can be examined from pairwise population-averaged cross-correlograms calculated from all pairs of the extracted units. We found that the activities are positively correlated for neurons of the same type (ON-ON, OFF-OFF), but negatively correlated for neurons of opposite type (i.e. ON-OFF). Also, the effect of different stimulus characteristics on the correlation is observed. While the correlation is decreased by conspecific-like stimuli, it is increased by prey-like stimuli. Furthermore, some neurons tend to fire synchronously at particular portions of stimulus, e.g. at specific phases of sinusoidal stimuli. Thus, this work will give important insights in how correlated activity contributes to the processing of natural stimuli.

